# Targeting Moonlighting Enzymes in Cancer

**DOI:** 10.3390/molecules29071573

**Published:** 2024-04-01

**Authors:** Chunxu Lin, Mingyang Yu, Ximei Wu, Hui Wang, Min Wei, Luyong Zhang

**Affiliations:** 1 Center for Drug Research and Development, Guangdong Pharmaceutical University, Guangzhou 510006, China; 2112140131@gdpu.edu.cn (C.L.); 2112040080@gdpu.edu.cn (M.Y.); 2112240081@gdpu.edu.cn (X.W.); 2112240063@gdpu.edu.cn (H.W.); 2 Jiangsu Key Laboratory of Drug Screening, China Pharmaceutical University, Nanjing 210009, China

**Keywords:** moonlighting enzyme, PTP4A3, targeted protein degradation, PROTAC

## Abstract

Moonlighting enzymes are multifunctional proteins that perform multiple functions beyond their primary role as catalytic enzymes. Extensive research and clinical practice have demonstrated their pivotal roles in the development and progression of cancer, making them promising targets for drug development. This article delves into multiple notable moonlighting enzymes, including GSK-3, GAPDH, and ENO1, and with a particular emphasis on an enigmatic phosphatase, PTP4A3. We scrutinize their distinct roles in cancer and the mechanisms that dictate their ability to switch roles. Lastly, we discuss the potential of an innovative approach to develop drugs targeting these moonlighting enzymes: target protein degradation. This strategy holds promise for effectively tackling moonlighting enzymes in the context of cancer therapy.

## 1. Introduction

Moonlighting proteins is the term used to describe a class of multifunctional proteins that perform more than one function within a cell, often without any change in their primary structure or sequence. The term “moonlighting” was coined by Dr. Constance Jeffery in 1999. In this seminal paper, Jeffery described moonlighting proteins as those that perform more than one function and do not have these functions as a consequence of gene fusions, families of homologous proteins, splice variants, or pleiotropic effects [1].

Since then, the comprehension of moonlighting proteins has undergone substantial development. The definition has been broadened to encompass proteins capable of transitioning between functions based on cellular conditions or executing distinct roles in various cellular compartments. A pivotal criterion for a protein to qualify as moonlighting has been elucidated: the autonomy of its diverse function, signifying that the disruption of one function (e.g., via mutation) should not influence the other function, and reciprocally. The notion of moonlighting proteins has gained widespread acceptance, as evidenced by a consistent influx of scholarly reviews focusing on this topic [2,3,4,5,6,7,8,9,10].

In the realm of enzymes, the term “moonlighting” pertains to proteins capable of executing multiple functions beyond their primary catalytic role. These proteins have evolved to encompass additional binding sites or functions while maintaining their original enzymatic activity. Enzymes stand out as compelling targets for drug development, with 50% of human protein drug targets belonging to the enzyme category [11]. They serve pivotal functions in diverse cellular processes and are intricately involved in disease pathways. Consequently, a significant number of current therapeutic approaches focus on inhibiting enzymes, with kinase and protease inhibitors being prominent examples that are widely utilized in clinical settings for the treatment of cancer.

Moonlighting enzymes challenge traditional views of protein specificity by showcasing how proteins can exhibit multiple functions beyond their primary roles. This versatility underscores the complexity of protein function and highlights the potential for developing novel therapeutic strategies targeting these moonlighting enzymes.

Moonlighting proteins are distributed across various species and can play crucial roles in disease development when their activities are altered. Moonlighting enzymes have been found to play critical roles in tumor growth and progression, making them potential targets for therapeutic intervention [6,9]. However, targeting moonlighting enzymes is challenging. Comprehensive understanding of the functions and regulations of moonlighting enzymes, and exploring innovative drug design strategies, are thus imperative for successful therapeutic intervention.

## 2. Moonlighting Enzymes Involved in Cancer

In this section, we aim to showcase specific instances of moonlighting enzymes that have been implicated in tumorigenesis. It is important to note that our selection represents only a subset of these enzymes, as previous reviews have provided comprehensive lists [6,9]. Additionally, we introduce a novel and emerging example, PTP4A3, which has not been extensively discussed in prior literature.

### 2.1. GSK-3

Glycogen synthase kinase 3 (GSK-3) is recognized as a serine/threonine kinase with a diverse range of substrates, including glycogen synthase, β-catenin, Cyclin D1, and c-Myc. These substrates are involved in important cellular processes, such as the cell cycle, apoptosis, and glycogen synthesis. The phosphorylation of glycogen synthase by GSK-3 inhibits its activity, thereby regulating glycogen metabolism [12,13]. Generally, the phosphorylation of GSK-3 substrates leads to the degradation of these proteins, which in turn results in the suppression of cell growth and proliferation [14,15].

Although GSK has been widely reported as a serine/threonine kinase, recent research has revealed it also functions as a scaffolding protein, involved in protein–protein interactions and organizing signaling complexes. One of the most well-studied examples of GSK-3 acting as a scaffolding protein is in the Wnt signaling pathway. GSK-3, Axin, adenomatous polyposis coli (APC), and casein kinase 1α (CK1α) forms a “destruction complex” without Wnt signaling, leading to the phosphorylation and subsequent degradation of β-catenin, a key effector of Wnt signaling. GSK-3 plays a crucial role in maintaining the stability of this complex, thereby enabling coordinated phosphorylation of β-catenin [14,16].

Furthermore, GSK-3 functions as a scaffold in the mTORC1 signaling pathway where it interacts with TSC2 (tuberous sclerosis complex 2) and PRAS40 (proline-rich Akt substrate of 40 kDa), two negative regulators of mTORC1. The interaction helps to inhibit mTORC1 activity under nutrient-poor conditions [17]. In addition to these findings, other studies have proved that GSK-3 can interact with various proteins in a kinase-independent manner, further demonstrating its role as a scaffolding protein [18].

The multifaceted roles of GSK-3 in the development of tumors and the progression of cancer make it a prime target for the development of new cancer treatments. Numerous GSK-3 inhibitors have demonstrated their potential in combating cancer through both pre-clinical and clinical studies [19,20]. One such example is Tideglusib, a non-ATP-competitive GSK-3β inhibitor. This inhibitor has been evaluated in xenograft and PDX murine models for a wide range of human cancers, including neuroblastoma [21], glioblastoma [22], osteosarcoma [23], prostate [24], pancreatic [25], and lung cancer [26].

TWS119 is another GSK-3β inhibitor and has demonstrated its potential in curtailing cell proliferation and triggering apoptosis in human alveolar rhabdomyosarcoma cells [27]. Moreover, it exhibits efficacy in effectively regulating the intricate processes of epithelial–mesenchymal transition (EMT) and cancer stem cell (CSC) properties in triple-negative breast cancer [28].

In the context of CAR-T cell therapy, GSK-3 inhibition has been found to enhance the persistence and antitumor activity of CAR-T cells. In a study in mantle cell lymphoma, the innovative CD19-CD22 bispecific CAR-T cells, when augmented with TWS119, displayed a remarkable reduction in the expression of exhaustion markers such as PD-1 and LAG3. The combination of bispecific CAR-T cells with TWS119 not only exhibits enhanced antitumor capabilities in vitro but also outperforms both the untreated control group and the groups treated with single CAR constructs in vivo studies. The combination of GSK-3 inhibitors with CAR-T cell therapy may provide a double therapeutic advantage by directly targeting the tumor cells and promoting a more effective immune response mediated by the engineered CAR-T cells [29].

### 2.2. GAPDH

GAPDH (glyceraldehyde-3-phosphate dehydrogenase) is a ubiquitous enzyme central to glycolysis and has garnered attention for its multifaceted role in cancer biology. GAPDH demonstrates both enzymatic and non-enzymatic functions, with both being intricately involved in cancer progression, invasiveness, and metastasis [30]. The enzymatic function of GAPDH is mainly observed in glycolysis, where it catalyzes the conversion of glyceraldehyde-3-phosphate to 1,3-bisphosphoglycerate. This function is important for cancer cells as it provides energy to support rapid proliferation and survival within the tumor microenvironment [31,32].

Furthermore, GAPDH’s non-enzymatic functions are also critical in cancer progression by influencing diverse cellular processes beyond metabolism. Studies have shown that GAPDH is involved in transcriptional regulation [33,34], DNA repair [35,36], apoptosis modulation [37,38,39], and intracellular trafficking [40,41]. For example, apoptotic stimuli can activate NO formation and lead to the S-nitrosylation of GAPDH. This modification inhibits GAPDH’s catalytic activity and enables it to bind to Siah, an E3-ubiquitin-ligase, which transports GAPDH to the nucleus. Inside the nucleus, GAPDH stabilizes Siah, allowing it to degrade specific target proteins and influence apoptosis. In the context of DNA repair, it is found that the activation of the tyrosine kinase Src occurs under DNA damage stress, leading to the phosphorylation of GAPDH at Tyr41. This phosphorylation event is crucial for the nuclear translocation of GAPDH. Nuclear GAPDH is recruited to DNA lesions and interacts with DNA polymerase β (Pol β) to participate in DNA repair mechanisms. Furthermore, nuclear GAPDH enhances Pol β polymerase activity and improves the efficiency of base excision repair (BER). GAPDH is also involved in apoptosis cascade in the aging process where the S-nitrosation of GAPDH, induced by iNOS, triggers its relocation to the nucleus. Once there, GAPDH actively mediates the process of apoptosis. These non-metabolic functions significantly promote tumorigenesis and tumor progression [31,32].

The dual roles of GAPDH in cancer are a good example of its ability to control cell death pathways. While some studies suggest GAPDH can increase cell apoptosis, others describe a protective function that promotes cell survival and tumor progression, which is extensively reviewed in [31]. Further exploration of the intricate interplay between GAPDH’s metabolic and non-metabolic functions is helpful for us to better understand how it is involved in tumor initiation and progression. This is essential for designing innovative therapeutic strategies targeting GAPDH.

### 2.3. ENO1

Enolase, known as phosphopyruvate hydratase, is another example of a moonlighting enzyme. Enolase catalyzes the conversion of 2-phosphoglycerate (2-PG) into phosphoenolpyruvate (PEP), the ninth step of glycolysis. Enolase is essential in energy metabolism and is expressed in all tissues and organisms [42,43].

There are three enolase subunits in humans: α, β, and γ, encoded by separate genes. The three subunits can combine into five isoenzymes. Among these, alpha-enolase (ENO1) plays a significant role in cancer progression, acting not only as an enzymatic protein, but also as a plasminogen receptor on the cell surface [44,45,46]. As a plasminogen receptor on the cell surface, ENO1 facilitates the conversion of plasminogen to plasmin, a potent proteolytic enzyme that degrades the extracellular matrix (ECM) and, thus, facilitates cell migration [45]. Studies have also shown that ENO1 expression correlates with poor prognosis of cancer patients, implicating a potential as a prognostic biomarker [45]. Furthermore, ENO1 participates in regulating integrin expression, which is a critical player in cancer cell adhesion, invasion, and metastasis [47]. The interaction between ENO1 and other proteins, such as protein arginine methyltransferase 5 (PRMT5), further exhibits its impact on cancer [48].

Overall, ENO1’s dual functions as an enzyme and plasminogen receptor underscore its significance in cancer progression and highlight its potential as a therapeutic target for cancer treatment.

### 2.4. PTP4A3

Another typical moonlighting protein falling into this category is PTP4A3, also known as protein tyrosine phosphatase 4A3 or PRL-3. It is a member of the protein tyrosine phosphatase (PTP) family. As its name suggests, PTP4A3 catalyzes the dephosphorylation of tyrosine or serine/threonine residues in protein molecules, playing a crucial role in cellular signaling transduction. Its role in promoting cancer initiation, metastasis, drug resistance, and recurrence has been validated in various cancer cell lines and animal models, with its high expression being inversely correlated with the prognosis of several solid tumors [49]. Several substrates of PTP4A3 have been reported, including FZR1 [50], Keratin 8 [51,52], Integrin β1 [52], and Leo l [53], where the phosphorylation levels of the substrates are regulated by PTP4A3, thereby affecting cancer-related signaling pathways and cancer progression.

On the other hand, there is substantial experimental evidence indicating that PTP4A3 functions as a “pseudophosphatase”. For instance, PTP4A3 can bind to CNNM4 (a membrane protein related to magnesium ion transport), inhibiting the efflux of Mg^2+^ and thereby impacting downstream energy metabolism pathways to promote tumorigenesis and progression [54]. The crystal structure of the PTP4A3-CNNM3 complex reveals that PTP4A3 acts as a “pseudophosphatase”; the critical amino acid residue C104 in the phosphatase active center of PTP4A3 binds to Asp426 of CNNM3, which is not phosphorylated [55,56].

In another study on breast cancer, it was found that PTP4A3 can induce the transformation of normal cancer cells into stem-like cells by competitively binding to MEF2A with HDAC4 (histone deacetylase 4), a process independent of PTP4A3’s phosphatase activity. The interaction between PTP4A3 and HDAC4 leads to the dissociation of HDAC4 from MEF2A and histones, affecting the deacetylation reaction. Maintaining high levels of acetylated MEF2A and histones upregulates the expression of the stem cell key transcription factor SOX2, ultimately inducing cancer cell transformation into stem-like cells. Researchers have defined this function of PTP4A3 as an adaptor protein [57].

Our research in acute T-cell lymphoblastic leukemia (T-ALL) has shown that although PTP4A3 can influence the phosphorylation levels of Src kinase [58], subsequent studies have revealed that its oncogenic role in T-ALL does not depend on its enzymatic activity. The interactions between PTP4A3 and proteins such as LCK and CD3 in T-ALL are also independent of its enzymatic activity (unpublished data). Increasing evidence suggests that PTP4A3 is a moonlighting protein, concurrently performing the functions of a phosphatase and an adaptor.

Like GSK-3, the scaffolding role of PTP4A3 operates independently of its enzymatic action. However, the exact mechanism by which PTP4A3 toggles between its phosphatase activity and its “moonlighting” protein function within the context of diverse tumor types remains an area of active investigation. The dominant function that underpins its oncogenic role in tumors is yet to be conclusively determined.

## 3. Regulatory Mechanisms Governing Functions of Moonlighting Enzymes

Moonlighting enzymes are regulated by various mechanisms to ensure proper function switching. Post-translational modifications (PTMs) play a crucial role in prompting moonlighting proteins to switch functions. Additionally, the location and timing of each protein activity are vital. Moonlighting proteins may perform distinct functions in various cellular locations such as the cytoplasm, nucleus, or cell membrane.

### 3.1. The Impact of Spatial Positioning on the Functions of Moonlighting Enzymes

The location of proteins indeed plays a significant role in influencing their functions, and this principle applies to moonlighting enzymes as well. They may perform different roles in various cellular locations, such as the cytoplasm, nucleus, or cell membrane [59].

For example, the subcellular location of GAPDH significantly influences its functions, which is extensively reviewed in [60,61]. To encapsulate, GAPDH exhibits multifaceted roles depending on its cellular location. In the cytosol, it primarily performs as a pivotal enzyme in the metabolic process of glycolysis, catalyzing the conversion of glyceraldehyde 3-phosphate into 1,3-bisphosphoglycerate. However, when GAPDH translocates to the nucleus, it participates in the orchestration of gene expression, apoptosis, and DNA repair. At the cell surface, GAPDH can act as a receptor for certain bacterial pathogens, aiding in their entry into the cell. Within mitochondria, GAPDH contributes to apoptosis by interacting with voltage-dependent anion channels.

Another typical example of how subcellular location tightly regulates moonlighting functions of enzyme p53. This well-known tumor suppressor performs its canonical role as a transcription factor in the nucleus. Mono-ubiquitinated p53 induced by low levels of Mdm2 moves to the cytoplasm, where it triggers apoptosis and inhibits autophagy. The detailed regulation is reviewed in [62].

### 3.2. Regulation via Post-Translational Modification (PTM)

Post-translational modifications (PTMs) play an indispensable role in regulating protein functions and their subcellular localization. This universal principle is true for the regulation of moonlighting proteins as well. The ensuing discussion will focus on several key PTMs that significantly contribute to the nuanced regulation of moonlighting protein functions.

#### 3.2.1. Phosphorylation

Phosphorylation is one of the most common PTMs. Phosphorylation of moonlighting proteins can modulate their activity levels or even switch between divergent functions. For example, serine residue phosphorylation of GAPDH inhibits its glycolytic function while enhancing nuclear translocation, where it regulates gene transcription [61].

In the case of the α-enolase isoform, phosphorylation of specific tyrosine residues in the protein promotes its interaction with plasminogen to cleave the activation peptide of plasminogen, leading to the conversion of plasminogen to plasmin, an enzymatic mediator implicated in extracellular matrix degradation and tissue remodeling [44]. These instances underscore phosphorylation’s dynamic regulatory potential over moonlighting proteins.

#### 3.2.2. Acetylation

Acetylation is a PTM modification adding an acetyl group to a protein, typically on lysine residues. Acetylation generally affects protein stability, protein–protein interactions, and subcellular localization, all of which are closely related to their activity. For instance, acetylation of Hsp90 at lysine residue 294 has been reported to enhance its interaction with transcription factors, such as p53, leading to altered gene expression patterns. This acetylation modification converts Hsp90 from a canonical chaperone to a transcriptional regulator [63].

#### 3.2.3. Ubiquitination

Ubiquitination is a PTM where a small protein, ubiquitin, is attached to target proteins by a cascade of E3 enzymatic reactions. Ubiquitination can occur at different residues on the target protein, including lysine (K) residues, as well as the amino terminus (N-terminus) of the protein. This modification can regulate protein function, localization, or protein–protein interactions. Ubiquitination of moonlighting proteins can regulate the balance between their distinct functions by targeting them for degradation or influencing their interactions with other proteins, for example, a well-known tumor suppressor, PTEN, whose primary role is a lipid phosphatase. However, PTEN also has a less-understood nuclear function, where it engages in maintaining chromosomal integrity [64]. The ubiquitination of PTEN plays a crucial role in regulating its cellular localization, and thus its function. Mono-ubiquitinated PTEN translocates to the nucleus where it plays a role in DNA repair and maintaining chromosomal stability. Meanwhile, non-ubiquitinated PTEN resides in the cytoplasm and acts as a phosphatase to negatively regulate the PI3K/Akt pathway [65].

#### 3.2.4. SUMOylation

Small ubiquitin-like modifier (SUMO) modification involves the attachment of SUMO proteins to target proteins. SUMOylation of moonlighting proteins can impact their functions by modulating their subcellular localization or interactions with other proteins. SUMOylation of p53 can switch its function from a transcription factor to a regulator of cellular metabolism. SUMOylation of p53 at specific lysine residues, such as K386 and K390, inhibits p53-dependent transcription by preventing its binding to DNA/chromatin [66]. When p53 is SUMOylated, it can translocate to the mitochondria and interact with proteins involved in metabolic pathways [66].

The intricate processes governing the regulation of moonlighting proteins’ diverse functionalities largely remain enigmatic; for instance, the subcellular location of PTP4A3 and its PTM are largely unknown. PTP4A3 belongs to a class of prenylated protein tyrosine phosphatases (PTPs) that are associated with the cell plasma membrane. Studies have shown that these PTPs are prenylated proteins in vivo, suggesting their localization at the cell membrane [67]. Therefore, research focusing on the subcellular location and post-translational modifications (PTMs) of moonlighting enzymes, and how they regulate multiple-function switching, are indeed valuable areas of exploration.

## 4. Targeting Moonlighting Enzymes for Cancer Treatment

There are two main strategies to effectively target multifunctional enzymes. The first is precise targeting, which blocks a specific function while leaving the others intact. Consider an enzyme that is important to both standard metabolic functions and anti-apoptotic pathways. If the purpose is to block the enzyme’s anti-apoptotic function without meddling with its metabolic role, then precise targeting would be the preferred choice.

The secondary strategy, termed as the “abolish-all” tactic, is designed to suppress all the enzyme’s functions. This method is particularly beneficial where every activity is hijacked to advance the disease. By simply turning off everything, we can nullify the enzyme’s multifunctionality and prevent all its harmful impacts in the disease progression.

In the realm of current drug design technologies, the strategy of precise targeting poses immense challenges. Therefore, our upcoming discussion will focus on the abolish-all strategy, which can be further subdivided into inhibiting the enzyme’s protein expression, inhibiting enzyme activity, or target protein degradation (TPD). TPD is a strategy that has emerged in the past two decades, representing a more thorough abolish-all approach. Hence, we will now delve into the potential of this strategy in targeting dual-function enzymes, using PTP4A3 as a case study.

### 4.1. Target Protein Degradation

The traditional method of drug discovery primarily involves directly modulating protein activity. The development and application of protein activity regulators, particularly inhibitors, have long been the mainstream of drug development. Over the past two decades, the technology of proteolysis targeting chimeras (PROTACs), which utilizes the body’s own protein clearance system to remove pathogenic target proteins, has been rapidly developing and has become a major weapon in new drug research and development. In addition to PROTACs, several new targeted protein degradation (TPD) strategies have emerged, including molecular glues, lysosome-targeting chimeras (LYTACs), and antibody-based PROTACs (AbTACs). TPD primarily utilizes the two major protein clearance systems in cells: the ubiquitin–proteasome system (UPS) and the lysosome. Most TPD strategies, such as PROTACs, molecular glues, and degradation tags (dTAGs), rely on the UPS and are mainly targeted at intracellular proteins. Lysosome-dependent TPD strategies can degrade membrane proteins, extracellular proteins, and protein aggregates, thereby greatly expanding the range of substrates. The history and current status of TPD development, as well as its application in drug discovery and design, have been summarized in numerous review articles [68,69,70,71,72,73,74,75].

The key difference between TPD, such as PROTAC technology, and traditional small molecule protein inhibitors/antibodies, is that PROTAC is “event driven”, as opposed to small molecules or antibodies which are “occupancy driven”. Occupancy driven implies that when small molecule inhibitors or antibodies inhibit a target protein’s activity, they need to occupy the protein’s active center for a long time. Small molecule inhibitors need to fulfill multiple requirements, including the following: a. high enough affinity to outcompete the target protein’s natural ligand/receptor; b. a large enough dose to saturate the target; and c. a long enough half-life to persistently inhibit protein activity. Therefore, the target protein needs a good drug pocket to allow the drug to bind firmly and regulate its activity [76].

In contrast, PROTAC only needs to briefly bind with the target protein/E3 enzyme, thereby triggering the degradation event and qualifying as “event driven”. It does not need to directly inhibit the function of the target protein, nor does it require sustained and intensive binding with the target protein. In theory, as long as the target protein has a ligand that can bind briefly, it can target proteins without suitable drug pockets or without an enzymatic active center. It has also been found in practice that PROTAC has a significant advantage in being able to transform specific poor target protein ligands into specific strong bifunctional molecules. For example, Foretinib is a pan-kinase inhibitor that can act on more than one hundred kinases, while two PROTAC molecules based on Foretinib can only degrade 14 and 9 kinases, respectively, greatly increasing their specificity. The reason for this might be that the interaction between target protein and E3 ubiquitin enzyme enhances selectivity [77,78].

### 4.2. Degrading PTP4A3 for Cancer Therapy

As discussed in Section 2.4, PTP4A3 is a recently discovered cancer target. Besides possessing traditional phosphatase functions, it also acts as a scaffolding protein or pseudophosphatase, and these functions are related to its cancer-promoting effects. The development of small molecule drugs targeting PTP4A3 faces multiple challenges. The enzyme active center, i.e., the binding cavity for small molecule compounds, is very flat and narrow, making it difficult to screen for high-affinity small molecule compounds [49]. Additionally, it is generally more difficult to screen for compounds that interfere with protein non-enzyme-promoting functions than to screen for compounds that inhibit its enzymatic activity.

Much effort has been put into developing PTP4A3 inhibitors. Various lead compound screening strategies, including high-throughput screening, virtual screening, and natural product screening, have been used in the research and development of PTP4A3 inhibitors. More than a dozen small molecule compounds that can inhibit the phosphatase activity of PTP4A3 in vitro have been discovered. These include JMS-053 [79], thienopyridone [80], analog 3 [81,82], rhodanine and its derivatives [83,84,85], and various natural products [49]. JMS-053, thienopyridone, analog 3, and rhodanine are widely used as tool drugs in the basic research of PTP4A3. However, most of these small molecule inhibitors have limited therapeutic effects or poor selectivity [49]. Therefore, no PTP4A3 small molecule inhibitors have entered clinical research yet.

Thus, a new horizon in overcoming the challenges of poor drug-like properties could be the development of PROTAC molecules that specifically target PTP4A3. This could pave the way for a new era of clinical drug development, specifically for the treatment of malignant tumors that are PTP4A3-dependent. We recently carried out a principle of concept study of developing a nanobody-based bio-PROTAC for the purpose of degrading PTP4A3. The preliminary study showed that this bio-PROTAC could degrade GFP-PTP4A3 fusion protein while sparing the homologous GFP-PTP4A1 or GFP-PTP4A2 (unpublished data). These preliminary research findings illuminate a path towards the targeted degradation of PTP4A3. We aim to evaluate the role of this bio-PROTAC in cancer.

## 5. Future Perspectives

It is now widely accepted that moonlighting is a universe nature of proteins, which is also true for enzymes. However, when moonlighting enzymes come under our scrutiny, particularly those with both enzymatic and non-enzymatic activities, we often neglect their non-enzymatic roles. Perhaps the exhaustive examination of their catalytic functions presented in the literature has generated a stereotype. However, such oversight could potentially pose limitations for drug design and development. For enzymes of this nature, it might be beneficial to consider an “abolish-all” approach, a strategy discussed above.

## Data Availability

No new data were created.

## References

[1] Jeffery C.J. (1999). Moonlighting proteins. Trends Biochem. Sci..

[2] Singh N., Bhalla N. (2020). Moonlighting Proteins. Annu. Rev. Genet..

[3] Jeffery C.J. (2016). Protein species and moonlighting proteins: Very small changes in a protein’s covalent structure can change its biochemical function. J. Proteom..

[4] Jeffery C.J. (2020). Enzymes, pseudoenzymes, and moonlighting proteins: Diversity of function in protein superfamilies. FEBS J..

[5] Huberts D.H.E.W., van der Klei I.J. (2010). Moonlighting proteins: An intriguing mode of multitasking. Biochim. Biophys. Acta-Mol. Cell Res..

[6] Min K.W., Lee S.H., Baek S.J. (2016). Moonlighting proteins in cancer. Cancer Lett..

[7] Jeffery C.J. (2018). Protein moonlighting: What is it, and why is it important?. Philos. Trans. R. Soc. B Biol. Sci..

[8] Espinosa-Cantú A., Ascencio D., Barona-Gómez F., De Luna A. (2015). Gene duplication and the evolution of moonlighting proteins. Front. Genet..

[9] Adamo A., Frusteri C., Pallotta M.T., Pirali T., Sartoris S., Ugel S. (2021). Moonlighting Proteins Are Important Players in Cancer Immunology. Front. Immunol..

[10] Jeffery C.J. (2019). An enzyme in the test tube, and a transcription factor in the cell: Moonlighting proteins and cellular factors that affect their behavior. Protein Sci..

[11] Bakheet T.M., Doig A.J. (2009). Properties and identification of human protein drug targets. Bioinformatics.

[12] Kazi A., Xiang S., Yang H., Delitto D., Trevino J., Jiang R.H.Y., Ayaz M., Lawrence H.R., Kennedy P., Sebti S.M. (2018). GSK3 suppression upregulates β-catenin and c-Myc to abrogate KRas-dependent tumors. Nat. Commun..

[13] Fang X., Yu S.X., Lu Y., Bast R.C., Woodgett J.R., Mills G.B. (2000). Phosphorylation and inactivation of glycogen synthase kinase 3 by protein kinase A. Proc. Natl. Acad. Sci. USA.

[14] Stamos J.L., Weis W.I. (2013). The β-catenin destruction complex. Cold Spring Harb. Perspect. Biol..

[15] Sears R., Nuckolls F., Haura E., Taya Y., Tamai K., Nevins J.R. (2000). Multiple Ras-dependent phosphorylation pathways regulate Myc protein stability. Genes. Dev..

[16] Kimelman D., Xu W. (2006). β-Catenin destruction complex: Insights and questions from a structural perspective. Oncogene.

[17] Inoki K., Li Y., Zhu T., Wu J., Guan K.L. (2002). TSC2 is phosphorylated and inhibited by Akt and suppresses mTOR signalling. Nat. Cell Biol..

[18] Beurel E., Grieco S.F., Jope R.S. (2015). Glycogen synthase kinase-3 (GSK3): Regulation, actions, and diseases. Pharmacol. Ther..

[19] Mathuram T.L., Reece L.M., Cherian K.M. (2018). GSK-3 Inhibitors: A Double-Edged Sword? An Update on Tideglusib. Drug Res..

[20] Augello G., Emma M.R., Cusimano A., Azzolina A., Montalto G., McCubrey J.A., Cervello M. (2020). The role of GSK-3 in cancer immunotherapy: GSK-3 inhibitors as a new frontier in cancer treatment. Cells.

[21] Bahmad H.F., Chalhoub R.M., Harati H., Bou-Gharios J., Assi S., Ballout F., Monzer A., Msheik H., Araji T., Elajami M.K. (2021). Tideglusib attenuates growth of neuroblastoma cancer stem/progenitor cells in vitro and in vivo by specifically targeting GSK-3β. Pharmacol. Rep..

[22] Bou-Gharios J., Assi S., Bahmad H.F., Kharroubi H., Araji T., Chalhoub R.M., Ballout F., Harati H., Fares Y., Abou-Kheir W. (2021). The potential use of tideglusib as an adjuvant radio-therapeutic treatment for glioblastoma multiforme cancer stem-like cells. Pharmacol. Rep..

[23] Wei D., Zhu X., Li S., Liu G., Wang Y., Wang W., Zhang Q., Jiang S. (2021). Tideglusib suppresses stem-cell-like features and progression of osteosarcoma by inhibiting GSK-3β/NOTCH1 signaling. Biochem. Biophys. Res. Commun..

[24] Sun A., Li C., Chen R., Huang Y., Chen Q., Cui X., Liu H., Thrasher J.B., Li B. (2016). GSK-3β controls autophagy by modulating LKB1-AMPK pathway in prostate cancer cells. Prostate.

[25] Hao Q., Gao L., Niu W., Chen L., Zhang P., Chen Z. (2020). POTEE stimulates the proliferation of pancreatic cancer by activating the PI3K/Akt/GSK-3β/β-catenin signaling. BioFactors.

[26] Daouk R., Hassane M., Bahmad H.F., Sinjab A., Fujimoto J., Abou-Kheir W., Kadara H. (2019). Genome-wide and phenotypic evaluation of stem cell progenitors derived from GPRC5A-deficient murine lung adenocarcinoma with somatic KRAS mutations. Front. Oncol..

[27] Zeng F.Y., Dong H., Cui J., Liu L., Chen T. (2010). Glycogen synthase kinase 3 regulates PAX3-FKHR-mediated cell proliferation in human alveolar rhabdomyosarcoma cells. Biochem. Biophys. Res. Commun..

[28] Vijay G.V., Zhao N., Den Hollander P., Toneff M.J., Joseph R., Pietila M., Taube J.H., Sarkar T.R., Ramirez-Pena E., Werden S.J. (2019). GSK3β regulates epithelial-mesenchymal transition and cancer stem cell properties in triple-negative breast cancer. Breast Cancer Res..

[29] Jin J., Li Y., Liu Y., Jordan A.A., McIntosh J., Vargas J., Che Y., Yao Y., Wang M. (2021). Bispecific CD19-CD20 and CD19-CD22 CAR-T Cells with Glycogen Synthase Kinase (GSK)-3β Inhibitor TWS119 Treatment Have Superior Therapeutic Effects on Mantle Cell Lymphoma. Blood.

[30] Sirover M.A. (2018). Pleiotropic effects of moonlighting glyceraldehyde-3-phosphate dehydrogenase (GAPDH) in cancer progression, invasiveness, and metastases. Cancer and Metastasis Reviews.

[31] Colell A., Green D.R., Ricci J.E. (2009). Novel roles for GAPDH in cell death and carcinogenesis. Cell Death Differ..

[32] Zhang J.Y., Zhang F., Hong C.Q., Giuliano A.E., Cui X.J., Zhou G.J., Zhang G.J., Cui Y.K. (2015). Critical protein GAPDH and its regulatory mechanisms in cancer cells. Cancer Biol. Med..

[33] Hara M.R., Snyder S.H. (2006). Nitric oxide-GAPDH-Siah: A novel cell death cascade. Cell. Mol. Neurobiol..

[34] Sun X.H., Lis J.T., Wu R. (1988). The positive and negative transcriptional regulation of the Drosophila Gapdh-2 gene. Genes. Dev..

[35] Kosova A.A., Khodyreva S.N., Lavrik O.I. (2017). Role of glyceraldehyde-3-phosphate dehydrogenase (GAPDH) in DNA repair. Biochemistry.

[36] Ci S., Xia W., Liang W., Qin L., Zhang Y., Dianov G.L., Wang M., Zhao X., Wu C., Alagamuthu K.K. (2020). Src-mediated phosphorylation of GAPDH regulates its nuclear localization and cellular response to DNA damage. FASEB J..

[37] Chen R.W., Saunders P.A., Wei H., Li Z., Seth P., Chuang D.M. (1999). Involvement of glyceraldehyde-3-phosphate dehydrogenase (GAPDH) and p53 in neuronal apoptosis: Evidence that GAPDH is upregulated by p53. J. Neurosci..

[38] Xie T., Qiao X., Sun C., Chu B., Meng J., Chen C. (2022). GAPDH S-nitrosation contributes to age-related sarcopenia through mediating apoptosis. Nitric Oxide.

[39] Hou X., Snarski P., Higashi Y., Yoshida T., Jurkevich A., Delafontaine P., Sukhanov S. (2017). Nuclear complex of glyceraldehyde-3-phosphate dehydrogenase and DNA repair enzyme apurinic/apyrimidinic endonuclease i protect smooth muscle cells against oxidant-induced cell death. FASEB J..

[40] Tisdale E.J., Talati N.K., Artalejo C.R., Shisheva A. (2016). GAPDH binds Akt to facilitate cargo transport in the early secretory pathway. Exp. Cell Res..

[41] Tisdale E.J., Kelly C., Artalejo C.E. (2004). Glyceraldehyde-3-phosphate dehydrogenase interacts with Rab2 and plays an essential role in endoplasmic reticulum to golgi transport exclusive of its glycolytic activity. J. Biol. Chem..

[42] Díaz-Ramos À., Roig-Borrellas A., García-Melero A., López-Alemany R. (2012). α-enolase, a multifunctional protein: Its role on pathophysiological situations. J. Biomed. Biotechnol..

[43] Didiasova M., Schaefer L., Wygrecka M. (2019). When place matters: Shuttling of enolase-1 across cellular compartments. Front. Cell Dev. Biol..

[44] Pancholi V. (2001). Multifunctional α-enolase: Its role in diseases. Cell. Mol. Life Sci..

[45] Almaguel F.A., Sanchez T.W., Ortiz-Hernandez G.L., Casiano C.A. (2021). Alpha-Enolase: Emerging Tumor-Associated Antigen, Cancer Biomarker, and Oncotherapeutic Target. Front. Genet..

[46] Song Q., Zhang K., Sun T., Xu C., Zhao W., Zhang Z. (2023). Knockout of ENO1 leads to metabolism reprogramming and tumor retardation in pancreatic cancer. Front. Oncol..

[47] Principe M., Borgoni S., Cascione M., Chattaragada M.S., Ferri-Borgogno S., Capello M., Bulfamante S., Chapelle J., Di Modugno F., Defilippi P. (2017). Alpha-enolase (ENO1) controls alpha v/beta 3 integrin expression and regulates pancreatic cancer adhesion, invasion, and metastasis. J. Hematol. Oncol..

[48] Zakrzewicz D., Didiasova M., Krüger M., Giaimo B.D., Borggrefe T., Mieth M., Hocke A.C., Zakrzewicz A., Schaefer L., Preissner K.T. (2018). Protein arginine methyltransferase 5 mediates enolase-1 cell surface trafficking in human lung adenocarcinoma cells. Biochim. Biophys. Acta Mol. Basis Dis..

[49] Wei M., Korotkov K.V., Blackburn J.S. (2018). Targeting phosphatases of regenerating liver (PRLs) in cancer. Pharmacol. Ther..

[50] Zhang C., Qu L., Lian S., Meng L., Min L., Liu J., Song Q., Shen L., Shou C. (2019). PRL-3 promotes ubiquitination and degradation of AURKA and colorectal cancer progression via dephosphorylation of FZR1. Cancer Res..

[51] Mizuuchi E., Semba S., Kodama Y., Yokozaki H. (2009). Down-modulation of keratin 8 phosphorylation levels by PRL-3 contributes to colorectal carcinoma progression. Int. J. Cancer.

[52] Peng L., Xing X., Li W., Qu L., Meng L., Lian S., Jiang B., Wu J., Shou C. (2009). PRL-3 promotes the motility, invasion, and metastasis of LoVo colon cancer cells through PRL-3-integrin β1-ERK1/2 and-MMP2 signaling. Mol. Cancer.

[53] Chong P.S.Y., Zhou J., Cheong L.L., Liu S.C., Qian J., Guo T., Sze S.K., Zeng Q., Chng W.J. (2014). LEO1 is regulated by PRL-3 and mediates its oncogenic properties in acute myelogenous leukemia. Cancer Res..

[54] Funato Y., Yamazaki D., Mizukami S., Du L., Kikuchi K., Miki H. (2014). Membrane protein CNNM4-dependent Mg2+ efflux suppresses tumor progression. J. Clin. Investig..

[55] Zhang H., Kozlov G., Li X., Wu H., Gulerez I., Gehring K. (2017). PRL3 phosphatase active site is required for binding the putative magnesium transporter CNNM3. Sci. Rep..

[56] Smith C.N., Kihn K., Williamson Z.A., Chow K.M., Hersh L.B., Korotkov K.V., Deredge D., Blackburn J.S. (2023). Development and characterization of nanobodies that specifically target the oncogenic Phosphatase of Regenerating Liver-3 (PRL-3) and impact its interaction with a known binding partner, CNNM3. PLoS ONE.

[57] Zhang M., Wei Y., Liu Y., Guan W., Zhang X., Kong J., Li H., Yang S., Wang H. (2020). Metastatic Phosphatase PRL-3 Induces Ovarian Cancer Stem Cell Sub-population through Phosphatase-Independent Deacetylation Modulations. iScience.

[58] Wei M., Haney M.G., Rivas D.R., Blackburn J.S. (2020). Protein tyrosine phosphatase 4A3 (PTP4A3/PRL-3) drives migration and progression of T-cell acute lymphoblastic leukemia in vitro and in vivo. Oncogenesis.

[59] Gupta M.N., Uversky V.N. (2023). Moonlighting enzymes: When cellular context defines specificity. Cell. Mol. Life Sci..

[60] Hildebrandt T., Knuesting J., Berndt C., Morgan B., Scheibe R. (2015). Cytosolic thiol switches regulating basic cellular functions: GAPDH as an information hub?. Biol. Chem..

[61] Tristan C., Shahani N., Sedlak T.W., Sawa A. (2011). The diverse functions of GAPDH: Views from different subcellular compartments. Cell. Signal..

[62] Lee J.T., Gu W. (2010). The multiple levels of regulation by p53 ubiquitination. Cell Death Differ..

[63] Scroggins B.T., Robzyk K., Wang D., Marcu M.G., Tsutsumi S., Beebe K., Cotter R.J., Felts S., Toft D., Karnitz L. (2007). An Acetylation Site in the Middle Domain of Hsp90 Regulates Chaperone Function. Mol. Cell.

[64] Shen W.H., Balajee A.S., Wang J., Wu H., Eng C., Pandolfi P.P., Yin Y. (2007). Essential Role for Nuclear PTEN in Maintaining Chromosomal Integrity. Cell.

[65] Trotman L.C., Wang X., Alimonti A., Chen Z., Teruya-Feldstein J., Yang H., Pavletich N.P., Carver B.S., Cordon-Cardo C., Erdjument-Bromage H. (2007). Ubiquitination Regulates PTEN Nuclear Import and Tumor Suppression. Cell.

[66] Wu S.Y., Chiang C.M. (2009). Crosstalk between sumoylation and acetylation regulates p53-dependent chromatin transcription and DNA binding. EMBO J..

[67] Wu S.Y., Chiang C.M. (2009). p53 sumoylation: Mechanistic insights from reconstitution studies. Epigenetics.

[68] Hu Z., Crews C.M. (2022). Recent Developments in PROTAC-Mediated Protein Degradation: From Bench to Clinic. ChemBioChem.

[69] Alabi S.B., Crews C.M. (2021). Major advances in targeted protein degradation: PROTACs, LYTACs, and MADTACs. J. Biol. Chem..

[70] Li K., Crews C.M. (2022). PROTACs: Past, present and future. Chem. Soc. Rev..

[71] Poso A. (2021). The Future of Medicinal Chemistry, PROTAC, and Undruggable Drug Targets. J. Med. Chem..

[72] Sakamoto K.M., Kim K.B., Kumagai A., Mercurio F., Crews C.M., Deshaies R.J. (2001). Protacs: Chimeric molecules that target proteins to the Skp1-Cullin-F box complex for ubiquitination and degradation. Proc. Natl. Acad. Sci. USA.

[73] Neklesa T.K., Winkler J.D., Crews C.M. (2017). Targeted protein degradation by PROTACs. Pharmacol. Ther..

[74] Békés M., Langley D.R., Crews C.M. (2022). PROTAC targeted protein degraders: The past is prologue. Nat. Rev. Drug Discov..

[75] Song J., Hu M., Zhou J., Xie S., Li T., Li Y. (2023). Targeted protein degradation in drug development: Recent advances and future challenges. Eur. J. Med. Chem..

[76] Pettersson M., Crews C.M. (2019). PROteolysis TArgeting Chimeras (PROTACs)—Past, present and future. Drug Discov. Today Technol..

[77] Cromm P.M., Samarasinghe K.T.G., Hines J., Crews C.M. (2018). Addressing Kinase-Independent Functions of Fak via PROTAC-Mediated Degradation. J. Am. Chem. Soc..

[78] Bondeson D.P., Smith B.E., Burslem G.M., Buhimschi A.D., Hines J., Jaime-Figueroa S., Wang J., Hamman B.D., Ishchenko A., Crews C.M. (2018). Lessons in PROTAC Design from Selective Degradation with a Promiscuous Warhead. Cell Chem. Biol..

[79] Lazo J.S., Isbell K.N., Vasa S.A., Llaneza D.C., Rastelli E.J., Wipf P., Sharlow E.R. (2023). Disruption of Ovarian Cancer STAT3 and p38 Signaling with a Small-Molecule Inhibitor of PTP4A3 Phosphatase. J. Pharmacol. Exp. Ther..

[80] Zhang Z., Kozlov G., Chen Y.S., Gehring K. (2019). Mechanism of thienopyridone and iminothienopyridinedione inhibition of protein phosphatases. Medchemcomm.

[81] Hoeger B., Diether M., Ballester P.J., Köhn M. (2014). Biochemical evaluation of virtual screening methods reveals a cell-active inhibitor of the cancer-promoting phosphatases of regenerating liver. Eur. J. Med. Chem..

[82] Park H., Jung S.K., Jeong D.G., Ryu S.E., Kim S.J. (2008). Discovery of novel PRL-3 inhibitors based on the structure-based virtual screening. Bioorg. Med. Chem. Lett..

[83] Min G., Lee S.K., Kim H.N., Han Y.M., Lee R.H., Jeong D.G., Han D.C., Kwon B.M. (2013). Rhodanine-based PRL-3 inhibitors blocked the migration and invasion of metastatic cancer cells. Bioorg. Med. Chem. Lett..

[84] Ahn J.H., Kim S.J., Park W.S., Cho S.Y., Ha J.D., Kim S.S., Kang S.K., Jeong D.G., Jung S.K., Lee S.H. (2006). Synthesis and biological evaluation of rhodanine derivatives as PRL-3 inhibitors. Bioorg. Med. Chem. Lett..

[85] Lin L., Lu L., Yuan C., Wang A., Zhu M., Fu X., Xing S. (2021). The dual inhibition against the activity and expression of tyrosine phosphatase PRL-3 from a rhodanine derivative. Bioorg. Med. Chem. Lett..

